# How to navigate the EU’s CBAM trilemma: A review of policy objectives and possible designs

**DOI:** 10.12688/openreseurope.22147.1

**Published:** 2026-01-02

**Authors:** Fausto Corvino

**Affiliations:** 1 Hoover Chair in Economic and Social Ethics & Institut supérieur de philosophie (ISP), Université catholique de Louvain, Louvain-la-Neuve, Walloon Region, 1348, Belgium

**Keywords:** carbon border adjustment mechanism, carbon leakage, climate justice, developing countries, European Union.

## Abstract

This article focuses on the trilemma faced by EU policymakers in balancing the three conflicting policy objectives of the EU's Carbon Border Adjustment Mechanism (CBAM). The first objective is to create a level playing field between EU and non-EU producers by setting a uniform carbon price across borders. The second objective is to uphold climate justice. The third objective is to encourage third countries to adopt their own carbon pricing measures thus extending the scope of a *de facto* climate club. The article reviews the three main approaches to navigating the CBAM trilemma and examines the resulting trade-offs between policy objectives. The first approach is the dual-track policy design favoured by EU institutions, in which any climate justice issues raised by the CBAM pass to the remit of foreign climate aid. The second approach involves setting differentiated CBAM prices for third countries, depending on their level of development, or granting some of them full exemptions from the policy. The third approach is to recycle CBAM revenues back to certain trading partners, particularly low- and lower-middle-income countries.

## Plain Language Summary

A Carbon Border Adjustment Mechanism (CBAM) is set to come into full effect in the EU in January 2026, with the first financial payments to be made retroactively one year later. Under the CBAM, EU operators must pay a tax when importing certain categories of goods from non-EU countries, to cover any difference in carbon pricing between those countries and the EU. The CBAM serves two main purposes: protecting EU companies from perceived ‘unfair’ competition from businesses in countries with less stringent climate policies and encouraging other countries to adopt ambitious climate policies — those that do so will have their exports exempted from the CBAM, either partially or fully. However, the EU and its Member States have also pledged to implement global climate action while taking into account the different historical responsibilities of countries for climate change and their respective capacities to act. Therefore, the implementation of the CBAM's two purposes risks conflicting with the climate fairness requirements that the CBAM must meet. This is because the CBAM could place a significant burden on low- and lower-middle-income countries that trade extensively with the EU, have limited opportunities for economic diversification, and are least responsible for the climate crisis that the CBAM aims to address. For these reasons, it can be said that the CBAM presents EU policymakers with a policy trilemma. This article analyses the trade-offs between the three competing objectives of CBAM, which are created by three potential design options. The first design is to implement the CBAM without exemptions, while providing additional climate aid to third countries. The second involves setting a lower CBAM price for certain vulnerable trading partners. The third consists of redistributing part of the CBAM revenues to low- and lower-middle-income countries.

## 1.  Introduction

This article provides a narrative review of the policy options available to EU policymakers to balance effectiveness and fairness in the implementation the EU's Carbon Border Adjustment Mechanism (CBAM). The CBAM is a tax on CO _2e_ emissions levied on specific imported goods at the EU border. These goods will henceforth be referred to as ‘CBAM goods’ and include cement, iron, steel, aluminium, fertilisers, electricity and hydrogen, as well as their relevant precursors, i.e. the raw materials used to produce the final goods ( European Union, 2023a). The aim of the CBAM is to ensure that the carbon price for CBAM goods produced in non-EU countries is as close as possible to the carbon price applied to the same type of goods in the EU ( Amendola, 2025; Erdogdu, 2025; Otto, 2025; Zhang *et al.*, 2024; Zhong & Pei, 2023). In fact, CBAM goods are considered to be at high risk of carbon leakage. This means that, if the carbon price faced by EU producers of these goods were to increase significantly compared to the price faced by producers in third countries, some production would likely shift from the EU to those countries ( Wettestad, 2023). The CBAM currently applies to direct emissions from production for all CBAM goods, as well as to indirect emissions resulting from the use of electricity during production, but only for certain goods such as cement and fertilisers ( European Union, 2023a, art. 7, para. 1). However, the EU Commission could potentially propose expanding the scope of the CBAM to include indirect emissions embedded in more, or even all, CBAM goods in the future ( European Union, 2023a, art. 30, para. 3).

The CBAM price applicable to a tonne of CO _2e_ emissions embedded in imports from a third country is determined by the difference, if any, between the EU's carbon price and that of the exporting country (or alternative levying mechanism). The EU carbon price fluctuates according to the supply and demand of emissions allowances within the EU Emissions Trading System (ETS), the EU's flagship cap-and-trade scheme ( Kotzampasakis & Woerdman, 2024). The EU ETS currently covers the power, heavy industry, aviation, and maritime sectors. Buildings and road transport will be included in a separate ETS2, which was initially scheduled for 2027 but has recently been postponed to 2028 ( European Parliament, 2025; European Union, 2023b). EU institutions set the supply of ETS allowances through an annual cap, which is reduced each year based on a linear reduction factor — the lower the factor, the greater the EU's mitigation effort. ETS allowances are purchased by registered operators, including companies and financial intermediaries, either at the weekly European Energy Exchange – EEX – auction platform or on the ETS secondary market, where operators can sell allowances that they have previously purchased or been allocated. As with any other commodity, if the supply of ETS allowances diminishes while demand remains constant or increases, the price of allowances increases. For a carbon price in a third country to be deducted from the applicable CBAM price, it must be levied directly on emissions produced during the manufacture of goods in that country. It must also form part of a direct levying scheme or a cap-and-trade system ( Boute, 2024; Ziyue, 2025).

The CBAM price applies to importers. Specifically, if an EU operator intends to import goods from a third country, they must first report the CO _2e_ emissions embedded in those goods. If the carbon price paid by local producers in the third country is lower than the EU carbon price, the EU operator must then purchase sufficient CBAM certificates to cover the carbon price difference. This places a double burden on third-country exporters. Firstly, they must implement the necessary measures to provide accurate data on the emissions embedded in their export products. Secondly, the fact that EU operators have to pay for CBAM certificates increases the cost of imported goods for EU consumers. This may put third-country producers under economic pressure to reduce production costs and/or profit margins in order to remain competitive in the EU market.

The CBAM was officially introduced in May 2023 as part of the EU's strategy for transitioning to a net-zero emissions economy by 2050, as set out in the 2019 EU Green Deal ( European Commission, 2019; European Union, 2023a). It entered its initial transitional phase in October 2023 ( Cornago & Berg, 2024; Erdogdu, 2025; Tamellini, 2024). During this phase, EU importers must account for and report the emissions embedded in goods crossing the EU border, but no payment is required. From January 2026, EU importers will be required to pay for the emissions embedded in the goods they import. However, some amendments to the CBAM regulation, proposed by the EU Commission as part of the Omnibus Simplification Package ( European Commission, 2025) and later approved by the European Parliament and the Council of the EU ( European Union, 2025), came into force in October 2025. Three main revisions have been made. Firstly, the sale of CBAM certificates will be postponed until January 2027. This does not extend the transition phase, as emissions produced in 2026 will still be paid for, albeit with a one-year delay. Secondly, a new annual import limit has been introduced below which EU operators are exempt from CBAM. Initially set at a monetary value of €150, the so-called *de minimis* exemption has now become a mass value of 50 tonnes. Thirdly, for countries without reliable data on emissions embedded in exports, default figures will be introduced based on data collected in other countries. Additionally, the EU Commission will publish default carbon prices for third countries with carbon taxation mechanisms in place but no general carbon price. No final decision has yet been made regarding the use of revenue generated by the CBAM. However, a separate EU Commission proposal on the EU budget suggests that Member States keep a quarter of the revenues, with the remaining three quarters going to the EU budget to repay the NextGenerationEU investment plan ( Marcu *et al.*, 2024, 1–2).

## 2.  Methods

This article presents the CBAM trilemma as a framework for understanding the challenges that EU policymakers face when taxing CO _2e_ emissions embedded in imports entering the EU. The trilemma arises from the need to balance three competing policy objectives. The first objective is to ‘level the playing field’ between EU and non-EU companies by reducing the difference in their carbon prices ( Bellora & Fontagné, 2023; Wettestad, 2025). The second objective is to promote global climate justice by fairly allocating the burdens and benefits of climate action between countries ( André & Pirlot, 2024; Corvino, 2025; Perdana & Vielle, 2022). The third objective is to encourage third countries to adopt their own carbon pricing measures and, potentially, their own CBAM, thereby enlarging the scope of a *de facto* climate club ( Overland & Huda, 2022; Szulecki *et al.*, 2022).

Section 3 of the article will analyse the normative and non-normative arguments in the literature supporting each of the three aforementioned policy objectives. Section 4 will examine the three main approaches to navigating the CBAM trilemma, as well as the trade-offs that these approaches create in relation to the policy's objectives. The first approach is to shift climate justice issues to the realm of foreign climate aid. The second approach is to renounce carbon price equivalence between EU and third countries and potentially grant exemptions from the CBAM to the most vulnerable countries. The third approach involves recycling CBAM revenues to low- and lower-middle-income countries, either in proportion to the carbon taxes they pay at the EU border or based on other criteria, with more or less conditional clauses attached.

The purpose of this article is twofold. Firstly, it aims to provide a comprehensive analysis of the advantages and disadvantages of the three main approaches to navigating the CBAM trilemma. This is relevant for EU policymakers in developing future amendments to the CBAM regulation, as well as for the research community and civil society when advocating for further reforms. Secondly, the article aims to highlight gaps in the existing literature on CBAM theory and practice and suggest possibilities for future research into additional policy options that could better help to achieve the policy objectives of the CBAM. As the article focuses on recent legislative developments concerning the CBAM, it draws on peer-reviewed literature, EU secondary legal sources, policy briefs by sector experts and recent opinion pieces by leading scholars.

## 3.  The CBAM trilemma

This section will provide an overview of the three objectives that give rise to the CBAM trilemma. The focus will be on justifying each objective and explaining how the CBAM can achieve them.

### 3.1  The level playing field objective

The first objective of the CBAM is to ensure that the carbon price faced by companies in third countries exporting certain goods to the EU is roughly equivalent to that applied to EU companies. To achieve this, the CBAM price levied on third countries should increase in line with the EU carbon price. This would prevent the EU's pursuit of the net-zero emissions goal from giving companies in other countries a competitive advantage over EU companies ( Bye *et al.*, 2025; Perdana & Vielle, 2025). There are at least three justifications for this.

The first argument concerns the effectiveness of climate mitigation measures ( Choudhury *et al.*, 2024; Fontagné & Schubert, 2023; Mehling *et al.*, 2019; Newman, 2022; Pirlot, 2024; Zhang, 2024). If EU companies were to face a competitive disadvantage as a result of a high EU carbon price, carbon leakage could ensue. This could occur either because EU companies would be incentivised to relocate production to third countries to avoid the carbon price, or because they would lose market share to non-EU producers operating under less stringent environmental regulations. Carbon leakage would render EU climate policy ineffective, with the victims of climate change worldwide bearing the brunt of the costs. The second argument is based on paternalism ( Gabbatiss & Song, 2024; Sagone & Cellura, 2025). Allowing low- and lower-middle-income countries to gain a competitive advantage based on unrestricted fossil fuel use would entrench them in outdated, energy-inefficient development models, which would harm them in the medium to long term. The argument relates to political feasibility ( Rodrik, 2022; Wettestad, 2025). Increasing the EU carbon price without protecting EU companies through the CBAM would likely lead to public backlash against EU climate policy.

Until now, the carbon price for EU heavy industry has been kept deliberately low. In some cases, it has even been negative. This has taken three forms. Firstly, unlike the EU power sector, EU heavy industry has received a significant number of ETS allowances free of charge, without having to participate in auctions. Currently, these free allowances cover over 90% of heavy industry emissions ( Sgaravatti, 2024). Notably, during certain periods, some companies received more ETS allowances for free than they needed overall. They have consequently been able to stockpile allowances and sell them on the secondary market at a profit ( Tamellini *et al.*, 2023). Secondly, the ETS allowance cap has been set with a deliberately low linear reduction factor — below 2% until 2021 and above 4% only from 2024 ( Wennick, 2024). Thirdly, EU countries have been permitted to provide state aid to companies facing cost increases due to the ETS, in the form of indirect cost compensation ( Ferrara & Giua, 2022). This has significantly diluted the financial incentives of the ETS. ^1^

In April 2023, the European Parliament and the Council of the EU adopted the ‘Fit for 55’ policy package. This included a reform of the EU ETS that aimed to introduce a genuine carbon price for heavy industry in the EU ( Oberthür & Kulovesi, 2025). The reform involves increasing the linear reduction factor for the annual cap on ETS allowances and phasing out indirect cost compensation. Most notably, it will lead to the gradual withdrawal of free ETS allowances between 2026 and 2034. The withdrawal of the free allowances will coincide with the introduction of the CBAM. Therefore, from 2026 onwards, the CBAM price will apply only to the proportion of emissions not covered by free ETS allowances in sectors where these are granted. Free allowances will be completely phased out by 2034, meaning by this time the CBAM levy will apply to 100% of emissions ( Tandon & Le Merle, 2024, 19).

### 3.2  The climate justice objective

By signing and ratifying the 2015 Paris Agreement, the EU and its Member States committed to implementing long-term decarbonisation strategies in accordance with the principle of common but differentiated responsibilities and respective capabilities — CBDR-RC ( UNFCCC, 2015, art. 4, para. 19). This principle is commonly understood to have two main implications ( Marin Duran & Scott, 2024; Peperkamp, 2022; Schneider, 2024). Firstly, developed countries must lead the way in taking action to mitigate climate change. Secondly, developing countries must receive aid and support from developed countries to ensure that their pursuit of climate action does not jeopardise their development prospects or the fulfilment of their populations' basic needs. For the CBAM to be compatible with the international climate justice commitments undertaken by the EU and its Member States, this policy must be designed fairly from a global perspective.

The fairness of a given climate policy can be analysed using either an isolationist or an integrationist approach to climate justice. The isolationist approach focuses on fairly distributing the benefits and burdens within the climate sphere — i.e. those related to climate mitigation, adaptation and compensation — without considering how benefits and burdens are distributed in other spheres, such as trade, migration, foreign direct investment and so on ( McLaughlin, 2024; Meyer & Roser, 2010; Roser & Seidel, 2016; Shue, 2015). In contrast, the integrationist approach holds that the distribution of the benefits and burdens of climate action must be analysed alongside the distribution of the benefits and burdens in any other available spheres. This is because looking at only one sphere would be short-sighted from the perspective of global justice ( André, 2025; Caney, 2012; Caney, 2018; Gosseries, 2023, 118–149).

From an isolationist perspective, the CBAM would be unfair if it imposed a lesser burden on EU countries than on countries that are less responsible for remedying climate change than EU countries, based on any reasonable principle of fair burden sharing. These are countries that have historically emitted much less per capita, benefited relatively less from fossil fuel–based economic growth and are significantly poorer than EU countries ( Heyward, 2021; Page, 2008). From an integrationist perspective, the CBAM is neither inherently fair nor unfair. Value judgements apply to the overall global distribution of benefits and burdens, rather than to the effect of a policy instrument in isolation. However, it could be argued that the CBAM would fall short of some minimum standards of global justice if it significantly disadvantaged a trading partner in meeting the basic needs of its population, and if this disadvantage could not be offset in other areas of distribution ( Caney, 2012, 291–293; Walton, 2020). One example of such a standard is the sufficiency principle, which states that everyone should have access to enough resources to meet a basic threshold for a decent life ( Schramme, 2024).

From either a climate isolationist or global integrationist perspective, a CBAM that equalises carbon prices across borders risks being unfair to CBAM-vulnerable countries. These are countries that meet the following three conditions ( Morone & Alfino, 2025; Perdana & Vielle, 2022; Tandon & Le Merle, 2024). Firstly, they have a carbon-intensive industrial infrastructure, meaning their industry emits relatively high levels of CO _2_ per unit of output. This increases the economic burden of complying with a carbon price equivalent to that in the EU. For example, the amount of CO _2e_ emitted to produce one unit of energy in Zimbabwe is over six times higher than in France ( Our World in Data, 2025). Therefore, a uniform carbon price across borders would result in much higher energy taxation in Zimbabwe than in France. Secondly, CBAM-vulnerable countries have limited public resources that they can use to diversify trade or mitigate the negative impact of the CBAM, including by introducing a domestic carbon price. Thirdly, CBAM-vulnerable countries rely heavily on trade with the EU. The most striking example is Mozambique, which exports 74% of its CBAM products to the EU. This accounts for around 7% of its GDP ( Economist Intelligence Unit, 2023). Examples of CBAM-vulnerable countries include North African nations such as Libya, Algeria, Tunisia and Egypt, which export fertilisers to the EU, as well as Morocco, which is vulnerable due to its electricity exports. Several sub-Saharan African countries, including Mozambique, Zambia, Nigeria, Cameroon and Ghana, are vulnerable due to their aluminium and steel exports. Similarly, Balkan countries such as Bosnia and Herzegovina, North Macedonia, Serbia and Montenegro are vulnerable due to their exports of goods such as electricity, cement and steel.

The literature typically addresses two primary methods of mitigating the risk of climate injustice that could be caused by the price equivalence sought by the CBAM. The first is to deviate partially from carbon price equivalence by granting CBAM-vulnerable countries a lower CBAM price than other countries, net of any possible deductions ( André & Pirlot, 2024; Magacho *et al.*, 2023; Sasmal *et al.*, 2024; Zhong & Pei, 2022). The second is to maintain full carbon price equivalence, while providing CBAM-vulnerable countries with the economic and technological resources needed to adapt to the CBAM ( Brandi, 2021; Corvino, 2025; European Union, 2023a: recital 74; Marcu *et al.*, 2024; Perdana & Vielle, 2022). This could be achieved by providing these countries with additional financial resources or by earmarking part of the CBAM revenues for redistribution to them.

### 3.3  The *de facto* climate club objective

The third objective of the CBAM is to incentivise the EU's trading partners to adopt their own domestic carbon pricing measures in response to the EU's CBAM, and, potentially, their own CBAM, in order to prevent the risk of carbon leakage towards third countries. This would extend the reach of a *de facto* climate club initiated by the EU. A climate club is formed when a group of countries coordinate the adoption of ambitious climate policies, such as establishing a minimum carbon price, and use incentives to encourage other countries to join ( Budak, 2025, 103–111; Huseby *et al.*, 2024). These incentives can be either positive, such as offering financial support, or negative, such as imposing tariffs on non-members. The idea is that if the club comprises enough countries, it can be structured so that it is more economically rational for non-members to join and align their climate mitigation policies with those of club members than to suffer exclusion ( Nordhaus, 2020). It has been argued that the EU has created a *de facto* climate club by adopting the CBAM ( Szulecki *et al.*, 2022). A climate club is considered *de facto* if it exhibits the characteristics of a climate club, despite lacking the multilateral component. The EU and its Member States have first set a regional carbon price and then introduced carbon price equivalence at the EU border for certain product categories. This incentivises other countries to adopt their own carbon pricing measures to exempt their exports from the EU's CBAM and then introduce their own CBAM to protect their companies from competition with those based in countries without a carbon price. It is expected that this will lead to the expansion of the *de facto* climate club pioneered by the EU ( Overland & Huda, 2022; Sejdiu, 2023; Tarr *et al.*, 2023). The advantage of a *de facto* climate club, compared to a classic climate club, is that it overcomes the political problem of multilateral initiation. It provides a valid alternative to the post-Paris climate regime, which has proved ineffective due to its consensual and voluntary nature ( Bernardo *et al.*, 2021; Pihl, 2020).

To illustrate how this mechanism would function at an international level, suppose that, once the CBAM is fully implemented, EU operators wish to import certain CBAM goods from a specific exporting country. We will consider two different scenarios. In the first scenario, the exporting country has not introduced a domestic carbon price. To import goods from this country, EU operators would also need to purchase sufficient CBAM certificates to cover the emissions embedded in those goods. This indirectly burdens operators in exporting countries too, as they may have to absorb part of the cost of CBAM certificates to remain competitive in the EU market ( Bayat *et al.*, 2025; Dobranschi *et al.*, 2024; Vu *et al.*, 2024). They may do this by cutting production costs or foregoing part of their profits ( Dolphin & Ferrucci, 2025; Magacho *et al.*, 2023). In the second scenario, by contrast, the exporting country has introduced a domestic carbon price, which may be equivalent to or lower than the EU's. Consequently, EU operators are exempt, either partially or fully, from purchasing CBAM certificates when buying goods from this country. Meanwhile, operators in the exporting country are not indirectly burdened by the CBAM. Clearly, introducing a domestic carbon price increases production costs in the exporting country, which places a burden on operators there that is probably no less than the CBAM burden in the first scenario. However, the fundamental difference between the two scenarios is that, while in the first scenario the EU collects CBAM revenues and can therefore decide to allocate them to its budget, as it appears to intend to do, in the second scenario the exporting country collects carbon price revenues, which can be used to offset the negative effects of carbon pricing on local operators. This could be achieved through lump-sum payments, tax cuts or carbon dividends, for example ( Mintz-Woo, 2024; Muth, 2023). Thus, the exporting country is incentivized to introduce a domestic carbon price rather than expose itself, albeit indirectly, to CBAM taxation at the EU border ( Cornago & Berg, 2024, 4).

How strong the incentive is for an exporting country to respond to the EU's CBAM with a domestic carbon price clearly depends on the intensity of its trade relations with the EU. The incentive may increase if the EU decides to expand the range of CBAM goods in the future ( European Parliament, 2022). In any case, if a trading partner introduces a domestic carbon price in response to the EU's CBAM, they will have reason to implement a CBAM of their own in order to protect local producers from competition with producers based in countries without a carbon price. This would, in turn, put pressure on other countries to adopt a carbon price and then protect their domestic industries with their own CBAM, for the aforementioned reasons. Overall, the result could be the progressive expansion of a *de facto* climate club via a domino effect.

## 4.  Navigating the CBAM policy trilemma

This section will briefly discuss the three main approaches available for balancing the conflicting objectives of the CBAM and the associated trade-offs.

### 4.1  The dual-track approach

The first approach to the CBAM trilemma involves EU countries seeking to achieve carbon price equivalence across EU borders without granting any exemptions. This is done while offsetting the potential burden of CBAM on vulnerable countries by committing to provide additional foreign climate aid. Within the framework of the United Nations Framework Convention on Climate Change – UNFCCC, this aid is referred to as international climate finance ( Gordon, 2023; Michaelowa & Sacherer, 2022; Shishlov & Censkowsky, 2022). This comprises the economic and technological resources that developed countries are legally required to provide to developing countries to assist with the implementation of climate mitigation and adaptation measures. This obligation is set out in the 2015 Paris Agreement ( UNFCCC, 2015, art. 9) and in decisions made at previous and subsequent COPs ( UNFCCC, 2009, art. 8; UNFCCC, 2024). This approach to the CBAM trilemma has been described as dual-track, as it essentially separates the climate justice objective of the CBAM from its policy design, treating the two as if they are on parallel tracks ( Corvino, 2025, 28–31). There is evidence that EU institutions are moving in this direction. The CBAM regulation recognises that low- and lower-middle-income countries should have access to the necessary resources to adapt to the regulation. These resources can be referred to as ‘CBAM offset’. However, the regulation specifies that the allocation of these resources will not be part of the CBAM mechanism but will instead be delegated to EU foreign climate aid policies ( European Union, 2023a, recital 74). This additional aid can be referred to as ‘extra climate aid’.

One might argue that the CBAM trilemma would be resolved if the value of ‘extra climate aid’ equalled that of the ‘CBAM offset’. This would enable the CBAM to achieve the level playing field and the *de facto* climate club objectives, while ensuring that vulnerable countries are not burdened by the policy. However, there are at least three reasons — two pragmatic and one normative — to resist this conclusion ( Corvino, 2025). Firstly, there is a risk of the two tracks moving at different speeds ( Shue, 2014, 27–26). Once the ‘CBAM offset’ shifts to the track of foreign climate aid and becomes ‘extra climate aid’, there is no guarantee that its deployment will proceed in parallel with the implementation of the CBAM. Indeed, EU countries have so far been reluctant to fulfil their obligations to provide foreign climate aid. Specifically, alongside other developed countries, they failed to honour their commitment made at COP15 in 2009 to mobilise $100 billion per year in international climate finance by 2020. Furthermore, much of the funding they have provided so far has been non-concessional ( Oxfam, 2023). At COP29 in 2024, the EU and other developed countries set a new climate finance target for the period after 2025 that falls far short of what the poorest countries need to cope with climate change ( UNEP, 2025). A further risk is double counting ( CARE Denmark, 2023, 16–19; Mitchell *et al.*, 2021; Weikmans *et al.*, 2017). The ‘extra climate aid’ should be both new and in addition to the foreign climate aid that EU countries are already committed to, regardless of the CBAM. Similarly, foreign climate aid should be new and in addition to what developed countries would or should provide to developing countries based on approved or planned projects and/or other international commitments. However, the difficulty of identifying a precise baseline means there is a risk that EU countries will simply end up reclassifying other planned or existing resource flows as ‘extra climate aid’.

Finally, there is the issue of weakened claims ( Corvino, 2025, 34–41). The CBAM is a unilateral, cross-border measure imposed by a coalition of stronger countries on a group of weaker countries, which could suffer significant economic setbacks as a result. This is an attempt by the former to tackle the climate crisis they have primarily caused. Consequently, CBAM-vulnerable countries have a compelling case for being provided with the ‘CBAM offset’. Providing the ‘CBAM offset’ takes precedence over the EU's other positive obligations of global justice, i.e. obligations stemming from the responsibility to assist rather than from the responsibility to avoid harming others and/or compensate for harm caused. However, when the ‘CBAM offset’ is added to the pool of foreign climate aid, the priority level of claims from CBAM-vulnerable countries for these resources decreases to the same level as claims from other potential recipients of foreign climate aid — i.e., countries that have not been directly harmed by the EU but which still have a claim to EU assistance, given their lower capacity to mobilise economic and technological resources for climate action. This issue of weakened claims might have significant practical implications if EU countries were to use domestic budgetary constraints to justify reducing their provision of foreign climate aid, for instance ( Engström, 2025; Heimberger, 2025).

### 3.2  The exemption approach

A second approach to the CBAM trilemma is to set a CBAM price lower than the EU carbon price for CBAM-vulnerable countries. This could satisfy climate justice requirements by easing the economic burden that the CBAM would otherwise impose on these countries. Full exemptions could even eliminate this burden entirely ( André & Pirlot, 2024; Magacho *et al.*, 2023; Sasmal *et al.*, 2024; Zhong & Pei, 2022). However, granting such exemptions would mean giving up full carbon price equivalence and limiting the scope of the climate club incentive. Whether and to what extent the consequences of abandoning carbon price equivalence are tolerable must be assessed in light of the three arguments for such equivalence set out previously.

Firstly, granting CBAM-vulnerable countries a lower carbon price than the EU could pose a risk of carbon leakage. Currently, there is no significant empirical evidence of carbon leakage from EU countries to CBAM-vulnerable countries ( Kittel & Fahl, 2025; Olasehinde-Williams & Akadiri, 2025). However, it could be argued that carbon leakage would ensue if the EU's carbon price were to escalate due to the recent ETS reform ( Jakob, 2021). This seems to be the main concern that has so far prompted EU institutions not to grant any exceptions to the CBAM ( Lowe, 2021). Therefore, the risk of carbon leakage associated with the exemption approach must be investigated further to quantify it more accurately, as well as the impact (if any) that granting exemptions would have on the attainment of the global climate mitigation targets set by the international community. Secondly, even if the risk of carbon leakage were limited, the exemption approach would reduce the incentive for CBAM-vulnerable countries to decarbonise their industries. Consequently, these countries could remain locked into fossil-based development models while the rest of the world transitions to more sustainable ones. This could have negative economic consequences for these countries in the medium to long term, and it could be opposed on paternalistic grounds ( Morone & Alfino, 2025, 8). Thirdly, the political feasibility of the exemption approach depends on its economic consequences for EU companies. This therefore ties in with the issue of more accurately quantifying the carbon leakage that could occur between the EU and certain low- and lower-middle-income countries if the latter are exempted from the CBAM. Finally, there is the question of how much of an obstacle the exemption approach poses to the *de facto* climate club objective. The exemption approach would provide little incentive for CBAM-vulnerable countries to align their climate policies with those of the *de facto* climate club launched by the EU. However, as these countries' economies are relatively small, their initial non-participation would probably not significantly impair the effectiveness or prospects of further expansion of the club.

### 3.3  The revenue-recycling approach

A third approach to the CBAM trilemma is to maintain carbon price equivalence without granting any exemptions, while also recycling some of the CBAM revenues back to trading partners. This could be done either in proportion to the CBAM taxes paid indirectly by third countries at the EU border, or according to their level of economic development ( Brandi, 2021; Corvino, 2025; Marcu *et al.*, 2024; Perdana & Vielle, 2022). In practice, this could mean that the largest proportion of CBAM revenues would be distributed to low- and lower-middle-income countries. The revenue-recycling approach would fulfil both carbon price equivalence and *de facto* climate club objectives. This is because the financial incentive of the CBAM for producers in these countries would remain, even if the revenues were recycled to their governments. However, to mitigate the risk of governments in third countries allocating the recycled revenues indiscriminately to domestic companies and thereby diluting the financial incentive of the CBAM, the recycling of these revenues could be made conditional upon a commitment to use them for climate action ( Cornago & Berg, 2024, 16).

The climate justice issue is more complex. Compared to the dual-track approach, the revenue-recycling approach has the advantage that the ‘CBAM offset’ would be guaranteed automatically for CBAM-vulnerable countries. However, the revenue-recycling approach may not allow enough leeway to prevent severe economic harm in CBAM-vulnerable countries, unlike the exemption approach. This is because the revenues generated at the EU border and returned to these countries would likely not fully offset the burden that the CBAM poses on their economies. Indeed, a potential drop in the production of CBAM goods in low- and lower-middle-income countries that are dependent on trade with the EU countries could have severe local consequences for workers, investors, the supply chain, and the welfare state ( Eicke *et al.*, 2021; Magacho *et al.*, 2023; Morone & Alfino, 2025). Moreover, the total revenue generated by CBAM is rather limited. It is estimated to amount to €1.5 billion per year from 2028 onwards ( Tamellini, 2024). Therefore, even if EU countries recycled revenues to CBAM-vulnerable countries in greater proportion to the CBAM taxes these countries pay at the EU border, it is unclear whether this would sufficiently offset the CBAM burden.

## 4.  Discussion

This narrative review of the EU's CBAM trilemma yields two main policy insights. First, exempting from the EU's CBAM those countries most exposed to its effects could be a satisfactory solution to the trilemma. However, this would only be the case if the carbon leakage that could result from this were to remain limited. Yet this is difficult to predict accurately in advance. This raises the question for policymakers of whether it is worth adopting the exemption approach by default and then correcting it if carbon leakage proves excessive. The answer depends on how policymakers balance their responsibility to prevent the worsening of the climate crisis against their responsibility towards CBAM-vulnerable countries. Conversely, the dual-track approach proposed by EU institutions, whereby the CBAM burden is offset for low- and lower-middle-income countries within the remit of foreign climate aid, leaves some climate justice issues unresolved. The adoption of this approach is not fully justified given the existence of the alternatives discussed in this article. Finally, the revenue-recycling approach, whereby at least some of the CBAM revenues are redistributed to CBAM-vulnerable countries, strikes a good balance between the two approaches discussed above. This approach could be adopted if the risks of carbon leakage materialise to the extent feared by EU policymakers.

The second policy insight is that the potential of hybrid approaches warrants further investigation. I will briefly mention two of these approaches. One possible approach is to recycle part of the CBAM revenues back to vulnerable trading partners in the form of ‘CBAM offset’, with any remaining uncompensated portion of the CBAM burden being addressed through ‘extra climate aid’. Further research is needed to determine the appropriate level of ‘extra climate aid’ required to achieve climate justice, as well as to mitigate the risk of CBAM implementation and foreign climate aid provision moving at different speeds. A second hybrid approach is to grant CBAM-vulnerable countries a temporary exemption from the CBAM, providing these countries with economic support from EU countries during this transition phase, either through revenue recycling or as ‘extra climate aid’. This transition phase would end once sufficient resources had been transferred to ensure that withdrawing the exemption would not cause injustice. These two hybrid approaches are excluded from this review as they are not sufficiently elaborated upon in the literature. However, the arguments analysed in this review suggest that these approaches warrant further investigation as they could provide an improved solution to the policy trilemma discussed thus far.

## 5.  Conclusions

The article provides a narrative review of the trilemma posed by the EU's CBAM in relation to its three main policy objectives, as well as the trade-offs between these objectives presented by the three most widely discussed policy designs. This is followed by a brief analysis of the results of the review and an identification of areas for further policy research.

## Ethics and consent

Ethical approval and consent were not required.

## Data Availability

No data associated with this article.
