# How to navigate the EU’s CBAM trilemma: A review of policy objectives and possible designs

**DOI:** 10.12688/openreseurope.22147.2

**Published:** 2026-04-16

**Authors:** Fausto Corvino

**Affiliations:** 1 Hoover Chair in Economic and Social Ethics & Institut supérieur de philosophie (ISP), Université catholique de Louvain, Louvain-la-Neuve, Walloon Region, 1348, Belgium

**Keywords:** carbon border adjustment mechanism, carbon leakage, climate justice, developing countries, European Union.

## Abstract

This article focuses on the trilemma faced by EU policymakers when balancing three conflicting policy goals of the EU in implementing the Carbon Border Adjustment Mechanism (CBAM). The first goal is to create a level playing field between EU and non-EU producers by setting a uniform carbon price across borders. The second goal is to uphold climate justice. The third goal is to encourage third countries to adopt their own carbon pricing measures thus extending the scope of a *de facto* climate club. The article reviews the four main approaches to navigating the CBAM trilemma and examines the resulting trade-offs between policy goals. The first approach, favoured by EU institutions, is the dual-track policy design, in which any climate justice issues raised by the CBAM pass to the remit of foreign climate aid. The second approach involves setting differentiated CBAM liabilities for third countries, depending on their level of development, or granting some of them full exemptions from the policy. The third approach is to recycle CBAM revenues back to low- and lower-middle-income countries. The fourth approach consists of enabling foreign operators to deduct voluntary carbon credits from their CBAM liabilities, thereby incentivising climate finance flows towards low- and lower-middle-income countries.

## Plain Language Summary

A Carbon Border Adjustment Mechanism (CBAM) came into full effect in the EU in January 2026, with the first financial payments to be made retroactively one year later. Under the CBAM, EU importers must pay a tax on certain categories of goods from non-EU countries to cover the difference in carbon pricing between the EU and those countries. The main objective of the CBAM is to shield EU companies from perceived 'unfair' competition from businesses based in countries with less stringent climate legislation than the EU. However, the CBAM could create a trade barrier that risks undermining the legitimate expectations of economic development of low- and lower-middle-income countries, which are, among other things, the least responsible for the climate crisis. This is in tension with the commitments made by the EU and its Member States in signing the 2015 Paris Agreement to advance collective climate mitigation efforts based on equity considerations. Finally, the EU regards the CBAM as an effective means of encouraging other countries to adopt climate mitigation policies aligned with its own. Such unilateral action could gradually lead to the formation of a so-called climate club. For all these reasons, it can be said that the CBAM presents EU policymakers with a policy trilemma. This article analyses the trade-offs between the EU's three competing goals in implementing the CBAM, arising from four alternative policy design options. The first design option would implement the CBAM without exemptions and provide additional climate aid to third countries. The second would introduce lower CBAM liabilities for certain vulnerable trading partners. The third proposes allocating part of the CBAM revenues to low- and lower-middle-income countries. The fourth aims to stimulate financial flows to these countries by allowing foreign operators to deduct certified carbon credits from their CBAM liabilities.

## 1. Introduction

This article provides a narrative review of the policy options available to EU policymakers to balance effectiveness and fairness in the implementation the EU’s Carbon Border Adjustment Mechanism (CBAM). The CBAM is a tax on CO _2e_ emissions levied on specific imported goods at the EU border. These goods will henceforth be referred to as ‘CBAM goods’ and include cement, iron, steel, aluminium, fertilisers, electricity and hydrogen, as well as their relevant precursors, i.e. the intermediate materials used in production ( European Union, 2023a). The aim of the CBAM is to ensure that the carbon price for CBAM goods produced in non-EU countries is as close as possible to the carbon price applied to the same type of goods in the EU ( Amendola, 2025; Erdogdu, 2025; Otto, 2025; Zhang *et al.*, 2024; Zhong & Pei, 2024). In fact, CBAM goods are considered to be at high risk of carbon leakage. This means that, if the carbon price faced by EU producers of these goods were to increase significantly compared to the price faced by producers in third countries, some production would likely shift from the EU to those countries ( Wettestad, 2023). The CBAM currently applies to direct emissions from production for all CBAM goods, as well as to indirect emissions resulting from the use of electricity during production, but only for certain goods such as cement and fertilisers ( European Union, 2023a, art. 7, para. 1). However, the EU Commission could potentially propose expanding the scope of the CBAM to include indirect emissions embedded in more CBAM goods, or even all CBAM goods, in the future ( European Union, 2023a, art. 30, para. 3; Stocchetti and Vassallo, 2025). Notably, in December 2025, the EU Commission proposed amendments to the CBAM regulation to extend the scope of the CBAM to certain downstream products that are steel/aluminium intensive − for example, industrial components, automotive parts, household appliances, etc. − starting in January 2028 ( European Commission, 2025a). The purpose of this measure is to prevent foreign manufacturers from circumventing the CBAM by exporting finished or semi-finished products to the EU instead of raw materials.

The CBAM liabilities applicable to a tonne of CO _2e_ emissions embedded in imports from a third country are determined by the difference, if any, between the EU’s carbon price and that of the exporting country, or alternative levying mechanism ( Sandler and Schrag, 2025, 2). The EU carbon price fluctuates according to the supply and demand of emissions allowances within the EU Emissions Trading System (ETS), the EU’s flagship cap-and-trade scheme ( Kotzampasakis & Woerdman, 2024). The EU ETS currently covers the power, heavy industry, aviation, and maritime sectors. Buildings and road transport will be included in a separate ETS2, which was initially scheduled for 2027 but has recently been postponed to 2028 ( European Parliament, 2025; European Union, 2023b). EU institutions set the supply of ETS allowances through an annual cap, which is reduced each year based on a linear reduction factor — the lower the factor, the greater the EU’s mitigation effort. ETS allowances are purchased by registered operators, including companies and financial intermediaries, either at the weekly European Energy Exchange – EEX – auction platform or on the ETS secondary market, where operators can sell allowances that they have previously purchased or been allocated. As with any other commodity, if the supply of ETS allowances diminishes while demand remains constant or increases, the price of allowances increases. For a carbon price in a third country to be deducted from CBAM liabilities, it must be part of a government-recognised carbon pricing mechanism, such as a direct carbon tax or a cap-and-trade system ( Boute, 2024; Wang, 2025; Sandler & Schrag, 2025, 8).

The CBAM price applies to importers. Specifically, if an EU operator intends to import goods from a third country, they must first report the CO _2e_ emissions embedded in those goods. If the carbon price paid by local producers in the third country is lower than the EU carbon price, the EU operator must then purchase sufficient CBAM certificates to cover the carbon price difference. This places a double burden on third-country exporters. Firstly, they must implement the necessary measures to provide accurate data on the emissions embedded in their export products. Secondly, the fact that EU operators have to pay for CBAM certificates increases the cost of imported goods for EU consumers. This may put third-country producers under economic pressure to reduce production costs and/or profit margins in order to remain competitive in the EU market.

The CBAM was officially introduced in May 2023 as part of the EU’s strategy for transitioning to a net-zero emissions economy by 2050, as set out in the 2019 EU Green Deal ( European Commission, 2019; European Union, 2023a). It entered its initial transitional phase in October 2023 ( Cornago & Berg, 2024; Erdogdu, 2025; Tamellini, 2024). During this phase, EU importers must account for and report the emissions embedded in goods crossing the EU border, but no payment is required. From January 2026, EU importers will be required to pay for the emissions embedded in the goods they import. However, some amendments to the CBAM regulation were proposed by the EU Commission as part of the Omnibus Simplification Package in February 2025 ( European Commission, 2025b) and came into force in October 2025 after approval from the European Parliament and the Council of the EU ( European Union, 2025). Three main revisions have been made. Firstly, the sale of CBAM certificates will be postponed until February 2027. This does not extend the transition phase, as emissions produced in 2026 will still be paid for, albeit with a one-year delay. Secondly, a new annual import limit has been introduced below which EU operators are exempt from CBAM. Initially set at a monetary value of €150 per consignment, the so-called *de minimis* exemption has now become a mass value of 50 tonnes per year. Thirdly, for countries without reliable data on emissions embedded in exports, default figures will be introduced based on data collected in other countries. Additionally, the EU Commission will publish default carbon prices for third countries with carbon taxation mechanisms in place but no general carbon price.

No final decision has yet been made regarding the use of revenue generated by the CBAM. However, the EU Commission has emphasised its intention to allocate these resources to the EU budget in order to repay, among other things, the NextGenerationEU investment plan, launched in 2020 to stimulate the European economy following the damage caused by the Covid-19 pandemic and primarily financed by common debt ( European Commission, 2023; Marcu *et al.*, 2024, 1–2). Moreover, in December 2025, the EU Commission proposed further amendments to the CBAM regulation to set up a ‘Temporary Decarbonisation Fund’ ( European Commission, 2025c). This measure aims to help EU energy-intensive companies offset any loss of competitiveness when operating in countries without an equivalent carbon pricing system to that of the EU. The proposal also specifies that the fund will be financed by Member States, with each contributing 25% of the CBAM revenues they collect in 2026 and 2027. Accordingly, it clarifies that the remaining 75% of CBAM revenues would constitute ‘a new Own Resource of the Union budget’ ( European Commission, 2025c, explanatory memorandum, 4–5; see also European Commission 2025d).

## 2. Methods

This article presents the CBAM trilemma as a framework for understanding the challenges that EU policymakers face when taxing the CO _2e_ emissions embedded in imports entering the EU. The trilemma arises from the need to balance three conflicting policy goals of the EU in implementing the CBAM. The first goal is to achieve the explicit objective of the CBAM regulation, which is to ‘level the playing field’ between EU and non-EU companies by reducing the difference in their carbon prices ( Bellora & Fontagné, 2023; Wettestad, 2025). The second goal is to promote global climate justice by fairly allocating the burdens and benefits of climate action between countries ( André & Pirlot, 2024; Corvino, 2025; Perdana & Vielle, 2022). The third goal is to encourage third countries to adopt their own carbon pricing measures and, potentially, their own CBAM, thereby enlarging the scope of a *de facto* climate club ( Overland & Huda, 2022; Szulecki *et al.*, 2022).

Section 3 of the article will analyse the normative and non-normative arguments in the literature supporting each of the three aforementioned policy goals. Section 4 will examine the four main approaches to navigating the CBAM trilemma, as well as the trade-offs that these approaches create in relation to the policy’s goal. The first approach is to shift climate justice issues to the realm of foreign climate aid. The second approach is to renounce carbon price equivalence between EU and third countries and potentially grant exemptions from the CBAM to the most vulnerable countries. The third approach involves recycling CBAM revenues to low- and lower-middle-income countries, either in proportion to the carbon taxes they pay at the EU border or based on other criteria, with more or less conditional clauses attached. The fourth approach consists of allowing foreign operators to deduct the monetary value of approved carbon credits resulting from emission reductions and/or negative emissions from their CBAM liabilities. This is expected to encourage investment in decarbonisation projects in developing countries, with the aim of generating carbon credits, from both foreign and domestic sources.

The purpose of this article is twofold. Firstly, it aims to provide a comprehensive analysis of the advantages and disadvantages of the four main approaches to navigating the CBAM trilemma. This is relevant for EU policymakers in developing future amendments to the CBAM regulation, as well as for the research community and civil society when advocating for further reforms. Secondly, the article aims to highlight gaps in the existing literature on CBAM theory and practice and suggest possibilities for future research into additional policy options that could better help to achieve the policy goals of the EU in the implementation of the CBAM. As the article focuses on recent legislative developments concerning the CBAM, it draws on peer-reviewed literature, EU secondary legal sources, policy briefs by sector experts and recent opinion pieces by leading scholars.

## 3. The CBAM trilemma

This section will provide an overview of the three EU policy goals that give rise to the CBAM trilemma. The focus will be on justifying each goal and explaining how the CBAM can achieve them.

### 3.1 The level playing field goal

The primary objective of the CBAM is to ensure that the carbon price faced by companies in third countries exporting certain goods to the EU is roughly equivalent to that applied to EU companies ( European Union, 2023a, recital 12). To achieve this, the CBAM price levied on third countries should increase in line with the EU carbon price. This would prevent the EU’s pursuit of the net-zero emissions target from giving companies in other countries a competitive advantage over EU companies ( Bye *et al.*, 2025; Perdana & Vielle, 2025). There are at least three justifications for this.

The first argument concerns the effectiveness of climate mitigation measures ( Choudhury *et al.*, 2024; Fontagné & Schubert, 2023; Mehling *et al.*, 2019; Newman, 2022; Pirlot, 2024; Zhang *et al.*, 2024). If EU companies were to face a competitive disadvantage as a result of a high EU carbon price, carbon leakage could ensue. This could occur either because EU companies would be incentivised to relocate production to third countries to avoid the carbon price, or because they would lose market share to non-EU producers operating under less stringent environmental legislation. Carbon leakage would render EU climate policy ineffective, with the victims of climate change worldwide bearing the brunt of the costs. The second argument is based on paternalism ( Gabbatiss & Song, 2024; Sagone & Cellura, 2025). Allowing low- and lower-middle-income countries to gain a competitive advantage based on unrestricted fossil fuel use would entrench them in outdated, energy-inefficient development models, which would harm them in the medium to long term. The third argument relates to political feasibility ( Rodrik, 2022; Wettestad, 2025). Increasing the EU carbon price without protecting EU companies through the CBAM would likely lead to public backlash against EU climate policy.

Until now, the carbon price for EU heavy industry has been kept deliberately low. In some cases, it has even been negative. This has taken three forms. Firstly, unlike the EU power sector, EU heavy industry has received a significant number of ETS allowances free of charge, without having to participate in auctions. Currently, these free allowances cover over 90% of heavy industry emissions ( Sgaravatti, 2024). Notably, during certain periods, some companies received more ETS allowances for free than they needed overall. They have consequently been able to stockpile allowances and sell them on the secondary market at a profit ( Tamellini *et al.*, 2023). Secondly, the ETS allowance cap has been set with a deliberately low linear reduction factor — below 2% until 2021 and above 4% only from 2024 ( Wennick, 2024). Thirdly, EU countries have been permitted to provide state aid to companies facing cost increases due to the ETS, in the form of indirect cost compensation ( Ferrara & Giua, 2022). This has significantly diluted the financial incentives of the ETS. ^1^

In April 2023, the European Parliament and the Council of the EU adopted the ‘Fit for 55’ policy package. This included a reform of the EU ETS that aimed to introduce a genuine carbon price for heavy industry in the EU ( Oberthür & Kulovesi, 2025). The reform involves increasing the linear reduction factor for the annual cap on ETS allowances and phasing out indirect cost compensation. Most notably, it will lead to the gradual withdrawal of free ETS allowances between 2026 and 2034. The withdrawal of the free allowances will coincide with the introduction of the CBAM. Therefore, from 2026 onwards, the CBAM liabilities will apply only to the proportion of emissions not covered by free ETS allowances in sectors where these are granted. Free allowances will be completely phased out by 2034, meaning by this time the CBAM liabilities will apply to 100% of emissions ( Tandon & Le Merle, 2024, 19). ^2^

### 3.2 The climate justice goal

By signing and ratifying the 2015 Paris Agreement, the EU and its Member States pledged to reduce global emissions within a collective framework grounded in considerations of equity ( UNFCCC, 2015, art. 14, para. 1). The equity of a given climate policy can be analysed using either an isolationist or an integrationist approach to climate justice ( McLaughlin, 2024). The isolationist approach focuses on equitably distributing the benefits and burdens within the climate sphere — i.e. those related to climate mitigation, adaptation and compensation — without considering how benefits and burdens are distributed in other spheres, such as trade, migration, foreign direct investment and so on ( Meyer & Roser, 2010; Roser & Seidel, 2016; Shue, 2015). In contrast, the integrationist approach holds that the distribution of the benefits and burdens of climate action must be analysed alongside the distribution of the benefits and burdens in any other available spheres. This is because looking at only one sphere would be short-sighted from the perspective of global justice ( André, 2025; Caney, 2012; Caney, 2018; Gosseries, 2023, 118–149).

From an isolationist perspective, the CBAM would be inequitable if it imposed a lesser burden on EU countries than on those that are less responsible for remedying climate change, based on any reasonable principle of fair burden sharing. These are countries that have historically emitted much less per capita, benefited relatively less from fossil fuel–based economic growth and are significantly poorer than EU countries ( Heyward, 2021; Page, 2008). All of these factors come under the umbrella of the principle of Common but Differentiated Responsibilities and Respective Capabilities (CBDR-RC), which is enshrined in the Paris Agreement ( Marin Duran & Scott, 2024; Peperkamp, 2022; Schneider, 2024). From an integrationist perspective, the CBAM is neither inherently fair nor unfair. Value judgements apply to the overall global distribution of benefits and burdens, rather than to the effects of a policy instrument in isolation. However, it could be argued that the CBAM would fall short of some minimum standards of global justice if it significantly disadvantaged certain trading partners in meeting the basic needs of their populations, and if this disadvantage could not be offset in other areas of distribution ( Caney, 2012, 291–293; Walton, 2020). One example of such a standard is the sufficiency principle, which states that each person should have access to enough resources to meet a basic threshold for a decent life ( Schramme, 2024).

The existence of two different approaches in the literature on climate justice raises the question of which should be used to evaluate the equity of the EU’s CBAM. Furthermore, both approaches risk being too broad and generic to provide actionable guidance on the CBAM. One way to operationalise these normative approaches is to argue that they both converge on the view that the CBAM is inequitable if it meets two conditions. Firstly, it prevents low- and lower-middle-income countries from trading with the EU. Secondly, such trade is crucial for the legitimate development prospects of these countries. This reflects the practical concerns voiced by partners in the Global South that the CBAM could hinder their continuity of trade with the EU ( Santer, 2025; Damborg *et al.*, 2026; Ottaviano, 2025; Dahl *et al.*, 2025). Moreover, if the CBAM is widely perceived as protectionist or discriminatory, it could become a geopolitical flashpoint. Low- and lower-middle-income countries could potentially join forces with emerging economies to oppose the cross-border policy in multilateral forums or even challenge its legality at the WTO ( Otto, 2025; Richard & Hahn, 2025; Marcu & Mehling, 2021).

More specifically, it could be argued that the EU’s CBAM is clearly inequitable if it prevents CBAM-vulnerable countries from trading with the EU. CBAM-vulnerable countries are those that meet the following three criteria ( Morone & Alfino, 2025; Perdana & Vielle, 2022; Tandon & Le Merle, 2024). Firstly, they have a carbon-intensive industrial infrastructure, meaning their industry emits relatively high levels of CO _2e_ per unit of output. This increases the economic burden of complying with a carbon price equivalent to that in the EU. For example, the amount of CO _2e_ emitted to produce one unit of energy in Zimbabwe is over six times higher than in France ( Our World in Data, 2025). Therefore, a uniform carbon price across borders would result in much higher energy taxation in Zimbabwe than in France. Secondly, CBAM-vulnerable countries have limited public resources that they can use to diversify trade or mitigate the negative impact of the CBAM, including by introducing a domestic carbon price. Thirdly, CBAM-vulnerable countries rely heavily on trade with the EU. The most striking example is Mozambique, which exports 74% of its CBAM products to the EU. This accounts for around 7% of its GDP ( Economist Intelligence Unit, 2023). Examples of CBAM-vulnerable countries include North African nations such as Libya, Algeria, Tunisia and Egypt, which export fertilisers to the EU, as well as Morocco, which is vulnerable due to its electricity exports. Several sub-Saharan African countries, including Mozambique, Zambia, Nigeria, Cameroon and Ghana, are vulnerable due to their aluminium and steel exports. Similarly, Balkan countries such as Bosnia and Herzegovina, North Macedonia, Serbia and Montenegro are vulnerable due to their exports of goods such as electricity, cement and steel.

The literature typically addresses two primary methods of mitigating the risk of climate injustice that could be caused by the price equivalence sought by the CBAM. The first is to deviate partially from carbon price equivalence by granting CBAM-vulnerable countries lower CBAM liabilities than other countries, net of any possible deductions ( André & Pirlot, 2024; Magacho *et al.*, 2024; Sasmal *et al.*, 2024; Zhong & Pei, 2022). The second is to maintain full carbon price equivalence, while providing CBAM-vulnerable countries with the economic and technological resources needed to adapt to the CBAM ( Brandi, 2021; Corvino, 2025; European Union, 2023a: recital 74; Marcu *et al.*, 2024; Perdana & Vielle, 2022). This could be achieved by providing these countries with additional financial resources or by earmarking part of the CBAM revenues for redistribution to them.

### 3.3 The *de facto* climate club goal

Although the EU’s CBAM is primarily intended to ensure carbon price equivalence across borders, the EU Commission and other EU institutions have repeatedly stated that they also see its implementation as a way of prompting other countries to adopt climate policies as stringent as the EU’s. This aligns with the CBAM regulation itself, which states that the EU’s CBAM aims to reduce the risk of carbon leakage ‘also by creating incentives for the reduction of emissions by operators in third countries’ ( European Union, 2023a, art. 1). Furthermore, the CBAM regulation states that: ‘The establishment of the CBAM calls for the development of bilateral, multilateral, and international cooperation with third countries. For that purpose, a forum of countries with carbon pricing instruments or other comparable instruments (‘Climate Club’) should be set up, in order to promote the implementation of ambitious climate policies in all countries and pave the way for a global carbon pricing framework’ ( European Union, 2023a, recital 72). The EU’s CBAM could serve this goal by prompting trading partners to introduce their own carbon pricing schemes, thereby exempting their exports from CBAM liabilities. If they do so, they may be further incentivised to implement their own CBAM to mitigate the risk of carbon leakage towards third countries. This would extend the reach of a *de facto* climate club initiated by the EU.

A climate club is formed when a group of countries coordinate the adoption of ambitious climate policies, such as establishing a minimum carbon price, and use incentives to encourage other countries to join ( Budak, 2025, 103–111; Huseby *et al.*, 2024). These incentives can be either positive, such as offering financial support, or negative, such as imposing tariffs on non-members. The idea is that if the club comprises enough countries, it can be structured so that it is more economically rational for non-members to join and align their climate mitigation policies with those of club members than to suffer exclusion ( Nordhaus, 2020). It has been argued that the EU has created a *de facto* climate club by adopting the CBAM ( Szulecki *et al.*, 2022). A climate club is considered to be *de facto* if it exhibits the characteristics of a climate club, despite lacking the multilateral component. The EU and its Member States have first set a regional carbon price and then introduced carbon price equivalence at the EU border for certain product categories. This incentivises other countries to adopt their own carbon pricing measures to exempt their exports from the EU’s CBAM and then introduce their own CBAM to protect their companies from competition with with producers based in countries without a carbon price. It is expected that this will lead to the expansion of the *de facto* climate club pioneered by the EU ( Overland & Huda, 2022; Sejdiu, 2023; Tarr *et al.*, 2023). The advantage of a *de facto* climate club, compared to a formal climate club, is that it overcomes the political problem of multilateral initiation. It provides a valid alternative to the post-Paris climate regime, which has proved ineffective due to its consensual and voluntary nature ( Bernardo *et al.*, 2021; Pihl, 2020).

To illustrate how this mechanism would function at an international level, suppose that, once the CBAM is fully implemented, EU operators wish to import certain CBAM goods from a specific exporting country. We will consider two different scenarios. In the first scenario, the exporting country has not introduced a domestic carbon price. To import goods from this country, EU operators would also need to purchase sufficient CBAM certificates to cover the emissions embedded in those goods. This indirectly burdens operators in the exporting country too, as they may have to absorb part of the cost of CBAM certificates to remain competitive in the EU market ( Bayat *et al.*, 2025; Dobranschi *et al.*, 2024; Vu *et al.*, 2024). They may do this by cutting production costs or foregoing part of their profits ( Dolphin & Ferrucci, 2025; Magacho *et al.*, 2024). In the second scenario, by contrast, the exporting country has introduced a domestic carbon price, which may be equivalent to or lower than the EU’s. Consequently, EU operators are exempt, either partially or fully, from purchasing CBAM certificates when buying goods from this country. Meanwhile, operators in the exporting country are not indirectly burdened by the CBAM. Clearly, introducing a domestic carbon price increases production costs in the exporting country, which places a burden on operators there that is probably no less than the CBAM burden in the first scenario. However, the fundamental difference between the two scenarios is that, while in the first scenario the EU collects CBAM revenues and can therefore decide to allocate them to its budget, as it appears to intend to do, in the second scenario the exporting country collects carbon price revenues, which can be used to offset the negative effects of carbon pricing on local operators. This could be achieved through lump-sum payments, tax cuts or carbon dividends, for example ( Mintz-Woo, 2024; Muth, 2023). Thus, the exporting country is incentivized to introduce a domestic carbon price instead of exposing its operators, albeit indirectly, to CBAM taxation at the EU border ( Cornago & Berg, 2024, 4).

How strong the incentive is for an exporting country to respond to the EU’s CBAM with a domestic carbon price clearly depends on the intensity of its trade relations with the EU. The incentive may increase if the EU decides to expand the range of CBAM goods in the future ( European Parliament, 2022). In any case, if a trading partner introduces a domestic carbon price in response to the EU’s CBAM, they will have reason to implement a CBAM of their own in order to protect local producers from competition with producers based in countries without a carbon price. This would, in turn, put pressure on other countries to adopt a carbon price and then protect their domestic industries with their own CBAM, for the aforementioned reasons. Overall, the result could be the progressive expansion of a *de facto* climate club via a domino effect.

## 4. Navigating the CBAM policy trilemma

This section will briefly discuss the four main approaches available for balancing the conflicting policy goals of the EU in designing the CBAM and the associated trade-offs.

### 4.1 The dual-track approach

The first approach to the CBAM trilemma involves EU countries seeking to achieve carbon price equivalence across EU borders without granting any exemptions. This is done while offsetting the potential burden of CBAM on vulnerable countries by committing to provide additional foreign climate aid. Within the framework of the United Nations Framework Convention on Climate Change – UNFCCC, this aid is referred to as international climate finance ( Gordon, 2023; Michaelowa & Sacherer, 2022; Shishlov & Censkowsky, 2022). This comprises the economic and technological resources that developed countries are legally required to provide to developing countries to assist with the implementation of climate mitigation and adaptation measures. This obligation is set out in the 2015 Paris Agreement ( UNFCCC, 2015, art. 9) and in decisions made at previous and subsequent COPs ( UNFCCC, 2009, art. 8; UNFCCC, 2024). This approach to the CBAM trilemma has been described as dual-track, as it essentially separates the climate justice goal from the policy design of the CBAM, treating the two as if they are on parallel tracks ( Corvino, 2025, 28–31). There is evidence that EU institutions are moving in this direction. The CBAM regulation recognises that low- and lower-middle-income countries should have access to the necessary resources to adapt to the regulation. These resources can be referred to as ‘CBAM offset’. However, the regulation specifies that the allocation of these resources will not be part of the CBAM mechanism but will instead be delegated to EU foreign climate aid policies ( European Union, 2023a, recital 74). This additional aid can be referred to as ‘extra climate aid’.

One might argue that the CBAM trilemma would be resolved if the value of ‘extra climate aid’ equalled that of the ‘CBAM offset’. This would enable the CBAM to achieve the level playing field and the climate club goals, while ensuring that vulnerable countries are not burdened by the policy. However, there are at least three reasons — two pragmatic and one normative — to resist this conclusion ( Corvino, 2025). Firstly, there is a risk of the two tracks moving at different speeds ( Shue, 2014, 27–26). Once the ‘CBAM offset’ shifts to the track of foreign climate aid and becomes ‘extra climate aid’, there is no guarantee that its deployment will proceed in parallel with the implementation of the CBAM. Indeed, EU countries have so far been reluctant to fulfil their obligations to provide foreign climate aid. Specifically, alongside other developed countries, they failed to honour their commitment made at COP15 in 2009 to mobilise $100 billion per year in international climate finance by 2020. Furthermore, much of the funding they have provided so far has been non-concessional ( Oxfam, 2023). At COP29 in 2024, the EU and other developed countries set a new climate finance target for the period after 2025 that falls far short of what the poorest countries need to cope with climate change ( UNEP, 2025). A further risk is double counting ( CARE Denmark, 2023, 16–19; Mitchell *et al.*, 2021; Weikmans *et al.*, 2017). The ‘extra climate aid’ should be both new and in addition to the foreign climate aid that EU countries are already committed to, regardless of the CBAM. Similarly, foreign climate aid should be new and in addition to what developed countries would or should provide to developing countries based on approved or planned projects and/or other international commitments. However, the difficulty of identifying a precise baseline means there is a concern that EU countries may simply end up reclassifying other planned or existing resource flows as ‘extra climate aid’.

Finally, there is the issue of weakened claims ( Corvino, 2025, 34–41). The CBAM is, among other things, a unilateral cross-border measure imposed by a coalition of stronger countries on a group of weaker countries, which could suffer significant economic setbacks as a result. This is an attempt by the former to tackle the climate crisis they have primarily caused. Consequently, CBAM-vulnerable countries have a compelling case for being provided with the ‘CBAM offset’. Providing the ‘CBAM offset’ takes precedence over the EU’s other positive obligations of global justice. These obligations stem from the responsibility to assist, rather than from the responsibility to avoid harming others and/or compensate for harm caused. However, when the ‘CBAM offset’ is added to the pool of foreign climate aid, the priority level of claims from CBAM-vulnerable countries for these resources decreases to the same level as claims from other potential recipients of foreign climate aid. This includes countries that have not been directly harmed by the EU, but which still have a claim to EU assistance, given their lower capacity to mobilise economic and technological resources for climate action. This issue of weakened claims might have significant practical implications if EU countries were to use domestic budgetary constraints to justify reducing their provision of foreign climate aid, for instance ( Engström, 2025; Heimberger, 2025).

### 4.2 The exemption approach

A second approach to the CBAM trilemma is to set a CBAM price lower than the EU carbon price for CBAM-vulnerable countries. This could satisfy climate justice requirements by easing the economic burden that the CBAM would otherwise impose on these countries. Full exemptions could even eliminate this burden entirely ( André & Pirlot, 2024; Magacho *et al.*, 2024; Sasmal *et al.*, 2024; Zhong & Pei, 2022). However, granting such exemptions would mean giving up full carbon price equivalence and limiting the scope of the climate club incentive. Whether and to what extent the consequences of abandoning carbon price equivalence are tolerable must be assessed in light of the three arguments for such equivalence set out previously.

Firstly, granting CBAM-vulnerable countries a lower carbon price than the EU could pose a risk of carbon leakage. Currently, there is no significant empirical evidence of carbon leakage from EU countries to CBAM-vulnerable countries ( Kittel & Fahl, 2025; Olasehinde-Williams & Akadiri, 2025). However, it could be argued that carbon leakage would ensue if the EU’s carbon price were to escalate due to the recent ETS reform ( Jakob, 2021). This seems to be the main concern that has so far prompted EU institutions not to grant any exceptions to the CBAM ( Lowe, 2021). Therefore, the risk of carbon leakage associated with the exemption approach must be investigated further to quantify it more accurately, as well as the impact (if any) that granting exemptions would have on the attainment of the global climate mitigation targets set by the international community. Secondly, even if the risk of carbon leakage were limited, the exemption approach would reduce the incentive for CBAM-vulnerable countries to decarbonise their industries. Consequently, these countries could remain locked into fossil-based development models while the rest of the world transitions to more sustainable ones. This could have negative economic consequences for CBAM-vulnerable countries in the medium to long term, and it could be opposed on paternalistic grounds ( Morone & Alfino, 2025, 8). Thirdly, the political feasibility of the exemption approach depends on its economic consequences for EU companies. This therefore ties in with the issue of more accurately quantifying the carbon leakage that could occur between the EU and certain low- and lower-middle-income countries if the latter are exempted from the CBAM. Finally, there is the question of how much of an obstacle the exemption approach poses to the climate club goal. The exemption approach would provide little incentive for CBAM-vulnerable countries to align their climate policies with those of the *de facto* climate club launched by the EU. However, as these countries’ economies are relatively small, their initial non-participation would probably not significantly impair the effectiveness or prospects of further expansion of the club.

### 4.3 The revenue-recycling approach

A third approach to the CBAM trilemma is to maintain carbon price equivalence without granting any exemptions, while also recycling some of the CBAM revenues back to trading partners. This could be done either in proportion to the CBAM taxes paid indirectly by third countries at the EU border, or according to their level of economic development ( Brandi, 2021; Corvino, 2025; Marcu *et al.*, 2024; Perdana & Vielle, 2022). In practice, this could mean that the largest proportion of CBAM revenues would be distributed to low- and lower-middle-income countries. The revenue-recycling approach would fulfil both carbon price equivalence and climate club goals. This is because the financial incentive of the CBAM for producers in these countries would remain, even if the revenues were recycled to their governments. However, to mitigate the risk of governments in third countries allocating the recycled revenues indiscriminately to domestic companies and thereby diluting the financial incentive of the CBAM, the recycling of these revenues could be made conditional upon a commitment to use them for climate action ( Cornago & Berg, 2024, 16).

The climate justice issue is more complex. Compared to the dual-track approach, the revenue-recycling approach has the advantage that the ‘CBAM offset’ would be guaranteed automatically for CBAM-vulnerable countries. However, the revenue-recycling approach may not allow enough leeway to prevent severe economic harm in CBAM-vulnerable countries, unlike the exemption approach. This is because the revenues generated at the EU border and returned to these countries would likely not fully offset the burden that the CBAM poses on their economies. Indeed, a potential drop in the production of CBAM goods in low- and lower-middle-income countries that rely heavily on trade with the EU could have severe local consequences for workers, investors, the supply chain, and the welfare state ( Eicke *et al.*, 2021; Magacho *et al.*, 2024; Morone & Alfino, 2025). Moreover, the CBAM is projected to generate a total of approximately €2.1 billion in annual revenues by 2030, which is relatively small ( European Commission, 2025b). Therefore, even if EU countries recycled revenues to CBAM-vulnerable countries in greater proportion to the CBAM taxes these countries pay at the EU border, it is unclear whether this would sufficiently offset the CBAM burden.

### 4.4 The credit-based approach

The fourth approach to the CBAM dilemma is to enable foreign operators to offset their CBAM liabilities by using certified carbon credits. This is expected to provide financial incentives that will increase climate finance for low- and lower-middle-income countries ( Sandler & Schrag, 2025). Foreign operators can generate carbon credits in two main ways: internationally and domestically. In the former, a foreign operator invests in emissions reduction, such as decarbonisation and/or carbon removal, by another entity, whether public or private, located in another country. In the second case, a foreign operator invests in emissions reduction domestically or within their own organisation. Under the credit-based approach, foreign operators must be able to submit their valid carbon credits to their respective governments for certification for the purposes of the CBAM mechanism. These operators should then be permitted to present this certification to EU border authorities in the same way as a domestic carbon price, in order to obtain a deduction from their CBAM liabilities. To make this work, it is obviously necessary to establish what makes carbon credits eligible for CBAM purposes and what does not. As far as the international channel is concerned, one possible solution would be to link the EU’s CBAM to Article 6 of the Paris Agreement, which establishes the rules for countries to trade emissions reductions with each other while avoiding the risk of double counting ( Sandler & Schrag, 2025, pp. 4–5; Rentería *et al.*, 2024; Rahut *et al.*, 2025). For the domestic channel, existing voluntary certification frameworks for permanent carbon removals, carbon farming, and carbon storage in products could be integrated into the EU’s CBAM ( Sandler & Schrag, 2025, pp. 8–9).

Legally speaking, there are two ways to implement the credit-based approach ( Sandler & Schrag, 2025, 8). The simpler option would be for the EU Commission to issue an act clarifying that the current wording of Article 3 of the CBAM regulation also encompasses carbon credits. Currently, Article 3 defines the carbon price paid in the country of origin of imported goods as ‘the monetary amount paid in a third country, under a carbon emissions reduction scheme, in the form of a tax, levy or fee or in the form of emission allowances under a greenhouse gas emissions trading system’ ( European Union, 2023a, art. 3). The other option would be to amend the current CBAM regulation to explicitly reference carbon credits as an effective carbon price. This would be more politically complex. It should also be stressed that the EU ETS does not recognise carbon credits for compliance. Therefore, the implementation of the credit-based approach would lead to a scenario in which only exporters, and not EU operators, could reduce their carbon price liabilities by means of carbon credits.

The credit-based approach does not undermine carbon price equivalence if the EU recognises certified carbon credits at their monetary value instead of in terms of CO _2e_ tonnage ( Sandler & Schrag, 2025, 6–7). More precisely, if carbon credits were recognised by the EU in terms of CO _2e_ tonnage, an EU operator that produces one unit of emissions and bears a certain economic burden due to the EU carbon price could suffer a competitive disadvantage compared to a foreign operator that has one unit of emissions embedded in its exports to the EU but deducts part of its CBAM liabilities by purchasing carbon credits at a relatively low cost per unit. Conversely, if the EU recognises carbon credits at their market value, the foreign operator that exports an emission unit will face the same economic burden as EU operators producing the same emission unit. Ideally, the real value of carbon credits in euros should be taken into account. Where this is difficult or impossible, as is often the case, carbon credits could be valued at their prevailing market price ( Sandler & Schrag, 2025, 7). Therefore, it can be concluded that the credit-based approach can be designed to fully achieve the goal of carbon price equivalence.

At the same time, allowing carbon credits to be used to offset CBAM liabilities could reduce the incentive for third countries to implement strict carbon pricing policies in response to the EU’s CBAM. However, this is largely a theoretical risk. In practice, it would be unrealistic for a country with significant trade exposure to the EU to cover all its CBAM liabilities with carbon credits. Nevertheless, enabling foreign operators to use carbon credits to offset their CBAM liabilities could still encourage a third-country government to implement a lower domestic carbon price than it otherwise would if local operators were unable to use carbon credits to achieve carbon price equivalence. Therefore, it can be preliminarily concluded that the credit-based approach could hinder the full achievement of the climate club goal to some extent, but not to a significant degree.

The credit-based approach leads to financial resources that would otherwise become CBAM revenues being retained and/or transferred to low- and lower-middle-income countries ( Sandler & Schrag, 2025, 5). On the one hand, developed or emerging countries would be encouraged to purchase carbon credits from low- and lower-middle-income countries at a relatively low unit cost to reduce their CBAM liabilities. Consequently, the money that developed or emerging countries would have paid at the EU border is instead channelled towards poorer countries as climate finance. On the other hand, operators in low- and lower-middle-income countries would be incentivised, for the same reasons, to generate carbon credits domestically instead of being taxed at the EU border. This transforms money that these countries would otherwise transfer to the EU into domestic investments for climate action. Therefore, the credit-based approach is a step forward in terms of climate justice, provided that it allocates additional financial resources to CBAM-vulnerable countries for decarbonisation measures, thereby reducing their exposure to the CBAM. However, it would be unrealistic to expect the credit-based approach to offset the entire economic burden placed on CBAM-vulnerable countries by the CBAM. This is because, although these countries have great potential to reduce emissions, the large-scale expansion of carbon credits faces economic, financial and institutional obstacles such as initial investment, registration and monitoring costs, and so on ( UNCTAD, 2024). Therefore, while the credit-based approach contributes to the goal of climate justice, it cannot fully achieve it alone due to structural constraints.

## 5. Discussion

This narrative review of the EU’s CBAM trilemma provides some key policy insights. If carbon leakage resulting from significant differences in carbon prices between the EU and CBAM-vulnerable countries were limited, the exemption approach would be an effective solution to the CBAM policy trilemma. Yet, accurately predicting future carbon leakage is challenging. This raises the question for policymakers of whether it would be prudent to adopt the exemption approach by default and then adjust it if carbon leakage were to prove excessive. The answer depends on how policymakers balance their responsibility to prevent the worsening of the climate crisis against their responsibility towards CBAM-vulnerable countries. However, if the risks of carbon leakage materialise to the extent feared by EU policymakers, the revenue-recycling approach would strike a good balance between the EU’s policy goals examined in this article. On the one hand, this approach would partially satisfy the goal of climate justice by mitigating some of the negative consequences of the CBAM for CBAM-vulnerable countries. The success of this approach in this regard depends on the fiscal potential of this policy instrument, which in turn hinges partly on its scope. On the other hand, this approach would not compromise the goals of carbon price equivalence or of the climate club. Conversely, the dual-track approach raises some serious concerns regarding climate justice. Given the existence of the viable alternatives discussed in this article, the adoption of this approach is not fully justified. Therefore, it is particularly concerning that the design of the EU’s CBAM is moving in this direction. Finally, the credit-based approach helps to achieve part of the climate justice goal without undermining the carbon price equivalence goal or compromising the climate club goal too much. Therefore, under any reasonable carbon leakage or fiscal capacity scenario, there are compelling reasons to implement this approach as a partial solution to the CBAM trilemma. It should be stressed that this approach could easily be combined with some of the other approaches referred to above.

Furthermore, the potential of hybrid approaches warrants further investigation. I will briefly mention two such approaches. One possible approach is to recycle part of the CBAM revenues back to vulnerable trading partners in the form of ‘CBAM offset’, addressing any remaining uncompensated portion of the CBAM burden through ‘extra climate aid’ and/or climate finance triggered by carbon credits. Further research is needed to determine the appropriate level of ‘extra climate aid’ and/or climate finance required to fully achieve the climate justice goal. It is also necessary to develop mechanisms that can prevent the risk of CBAM implementation and foreign climate aid provision moving at different speeds. A second hybrid approach is to temporarily exempt CBAM-vulnerable countries from the CBAM, providing them with economic support from EU countries, either through revenue recycling or as ‘extra climate aid’. This transition phase would end once sufficient resources had been transferred to these countries such that withdrawing the exemption would not cause injustice. These two hybrid approaches are excluded from this review as they are not sufficiently elaborated upon in the literature. However, the arguments analysed in this review suggest that these approaches warrant further investigation as they could provide improved solutions to the policy trilemma discussed thus far.

Finally, it should be emphasised that the policy trilemma described in this article may evolve as circumstances change. Expanding the scope of the EU’s CBAM could certainly bolster the carbon price equivalence and climate club goals. However, it could also undermine the climate justice goal to such an extent that managing the trade-offs between these two types of goal could become challenging. For instance, extending the scope of the CBAM to include the indirect emissions of products that are currently only taxed for their direct emissions would place an extremely heavy administrative and economic burden on low- and lower-middle-income countries that have relatively energy-intensive industries ( Magacho *et al.*, 2024; Xiaobei *et al.*, 2022). In this scenario, both the revenue-recycling and credit-based approaches could be ineffective, making the exemption approach necessary to avoid placing excessive trade risks on these countries. Conversely, extending the scope of the CBAM to some downstream products, as recently proposed by the EU Commission ( European Commission, 2025a) would primarily affect emerging economies and developed countries ( Bonnet *et al.*, 2026). Among other things, it would increase CBAM revenues, which could be used within the revenue-recycling approach or hybrid variations to reduce the CBAM burden on low- and lower-middle-income countries. More in-depth normative analysis would be required to determine whether and under what conditions a redistribution of economic resources from the former to the latter countries is justified.

## 6. Conclusions

The article provides a narrative review of the trilemma posed by the EU’s implementation of the CBAM in relation to three main policy goals. It does this by analysing the trade-offs between these policy goals, as they emerge from the four most widely discussed policy designs of the CBAM. This is followed by a brief analysis of the review’s results and an identification of areas for further policy research.

## Ethics and consent

Ethical approval and consent were not required.

## Data Availability

No data associated with this article.

## Footnotes

^1^
This is also the reason why the EU ETS has been largely unsuccessful thus far. Since its introduction in 2005, emissions in ETS sectors have fallen by 38%. However, almost all of this reduction has been achieved by the energy production sector, which has diversified its energy sources and increased its efficiency. In contrast, heavy industry has only reduced its emissions by 9% in the same period ( Tamellini *et al.*, 2023, 11).

^2^
However, it should be noted that, according to reports in the press, EU industries lobbied EU institutions between the second half of 2025 and the first half of 2026 to delay or even block the phase-out of free ETS allowances ( Abnett, 2026). Some Member State governments have done the same ( Gupte, 2025). As Jeanne Marullaz (2025) argues, supporting the EU’s CBAM while opposing the gradual removal of free ETS allowances could suggest protectionist intentions.
