# Supplementary material for: How to navigate the EU’s CBAM trilemma: A review of policy objectives and possible designs
Source: Open Res Eur. 2026 Apr 16;6:5. Originally published 2026 Jan 2. [Version 2] doi: 10.12688/openreseurope.22147.2 (PMC13109700; doi:10.12688/openreseurope.22147.2)
Supplement: Peer review history

# Reviewer report: Reviewer response for version 2

**DOI:** 10.21956/openreseurope.25334.r72440

**Published:** 2026-04-24

**Authors:** Ely Sandler

**Affiliations:** 1 Harvard Kennedy School, Cambridge, Massachusetts, USA

I would approve this now with the revisions. Thank you.

Are all factual statements correct and adequately supported by citations?

Yes

Is the review written in accessible language?

Yes

Are the conclusions drawn appropriate in the context of the current research literature?

Yes

Is the topic of the review discussed comprehensively in the context of the current literature?

Yes

Reviewer Expertise:

NA

I confirm that I have read this submission and believe that I have an appropriate level of expertise to confirm that it is of an acceptable scientific standard.

# Reviewer report: Reviewer response for version 2

**DOI:** 10.21956/openreseurope.25334.r72439

**Published:** 2026-04-17

**Authors:** Weimin Zhang, Congyi Dai

**Affiliations:** 1 Earth and Environment, Boston University, Boston, Massachusetts, USA; 2 School for Environment and Sustainability, University of Michigan (Ringgold ID: 151791), Ann Arbor, Michigan, USA

Did a good job! I have no further comments to make.

Are all factual statements correct and adequately supported by citations?

Yes

Is the review written in accessible language?

Yes

Are the conclusions drawn appropriate in the context of the current research literature?

Yes

Is the topic of the review discussed comprehensively in the context of the current literature?

Partly

Reviewer Expertise:

Energy Justice, Energy Transition, Industrial Decarbonization, Policy Analysis

We confirm that we have read this submission and believe that we have an appropriate level of expertise to confirm that it is of an acceptable scientific standard.

# Reviewer report: Reviewer response for version 1

**DOI:** 10.21956/openreseurope.23975.r67582

**Published:** 2026-01-19

**Authors:** Weimin Zhang, Congyi Dai

**Affiliations:** 1 Earth and Environment, Boston University, Boston, Massachusetts, USA; 2 School for Environment and Sustainability, University of Michigan (Ringgold ID: 151791), Ann Arbor, Michigan, USA

This article offers a clear and policy-relevant narrative review of the emerging literature on the EU’s Carbon Border Adjustment Mechanism (CBAM). The proposed “trilemma” framework provides a useful heuristic for organizing ongoing debates around competitiveness, climate justice, and international policy coordination. That said, I have several suggestions that I believe would strengthen the conceptual precision and analytical completeness of the article, particularly given its aim to provide a comprehensive review of CBAM design options.

 1. Clarifying the attribution of policy objectives

 One point that would benefit from further clarification concerns the attribution of policy objectives. Throughout the article, the three elements of the “trilemma” are presented as objectives of the CBAM itself. Conceptually, however, it may be more precise to distinguish between:

 CBAM as a policy instrument, whose primary and legally explicit objective is to address carbon leakage and maintain a level playing field; and

 Broader EU climate and trade objectives, such as advancing climate justice or fostering a de facto climate club, which shape the political context and design choices surrounding CBAM.

 Reframing the trilemma as reflecting EU policy objectives in the implementation of CBAM, rather than intrinsic objectives of CBAM as such, would improve conceptual clarity without undermining the core analytical structure of the paper. This distinction could be reflected in the title, abstract, and selected passages in Sections 2 and 3.

 2. Broadening the framing of the “climate justice” objective

 The discussion of climate justice is thoughtful and well grounded in normative literature, particularly through the isolationist versus integrationist distinction. At the same time, the framing may appear somewhat narrow relative to the policy concerns of many low- and lower-middle-income trading partners.

 Beyond distributive justice considerations in a strict normative sense, concerns about trade continuity, political acceptability, and development trajectories play a central role in shaping third-country responses to CBAM. The analysis could be strengthened by explicitly acknowledging that what is framed as a “justice” objective also encompasses broader political economy considerations related to sustaining economic participation in EU-centered value chains.

 3. Engaging with credit-based or offset-linked design proposals

 To further enhance the comprehensiveness of the review, I encourage the author to engage with emerging proposals that conceptualize credit-based approaches as an alternative way of navigating the CBAM trilemma. In particular, recent literature has explored the possibility of allowing exporters from developing countries to retire high-integrity carbon credits (for example, under Article 6 mechanisms) in order to qualify these payments as a “carbon price effectively paid” for CBAM purposes.

 Such approaches differ analytically from the three design paths discussed in Section 4, as they potentially combine carbon price equivalence with climate finance generation, while avoiding explicit exemptions or reliance on external aid channels. Even if the author ultimately finds these proposals normatively or practically limited, briefly situating them within the comparative landscape would strengthen the article’s claim to provide a comprehensive review of current CBAM design debates.

 4. Minor suggestions

 The comparative discussion in Section 4 could benefit from more explicit articulation of the conditions under which one design approach might dominate another (e.g., assumptions about carbon leakage risks or fiscal capacity).

 A short concluding paragraph reflecting on how the trilemma might evolve as CBAM coverage expands or as more countries adopt domestic carbon pricing could further enhance the forward-looking relevance of the paper.

 As a minor technical point, I noticed that one reference is cited as “(Ziyue, 2025)”. In this case, “Wang” appears to be the author’s family name, with “Ziyue” being the given name. It may therefore be more appropriate to cite this work as “(Wang, 2025)” for consistency with standard referencing conventions.

Are all factual statements correct and adequately supported by citations?

Yes

Is the review written in accessible language?

Yes

Are the conclusions drawn appropriate in the context of the current research literature?

Yes

Is the topic of the review discussed comprehensively in the context of the current literature?

Partly

Reviewer Expertise:

Energy Justice, Energy Transition, Industrial Decarbonization, Policy Analysis

We confirm that we have read this submission and believe that we have an appropriate level of expertise to confirm that it is of an acceptable scientific standard, however we have significant reservations, as outlined above.

# Response

**Published:** 2026-03-09

**Authors:** Fausto Corvino

**Affiliations:** Hoover Chair in Economic and Social Ethics & Insti, Universite catholique de Louvain, Louvain-la-Neuve, Walloon Region, Belgium

Dear Weimin Zhang and Congyi Dai,

 Thank you very much for your valuable suggestions, which have helped me greatly in working on a second version of the article. Please find below a point-by-point response to your comments. Please note that, as some of the issues raised in reports 1 and 2 are similar, some of the responses below are repeated in my responses to report 1 and vice versa. This is unavoidable as I am not allowed to provide a general response to the two reports received so far; I can only respond to them individually.

 Point 1. In the new version, I have clarified the policy objectives of the CBAM as you suggested. Throughout the new version, I refer to EU policy goals in the implementation of the CBAM rather than CBAM objectives. I have also clarified that the explicit objective of the CBAM regulation is carbon price equivalence, and that the other goals mainly derive from the EU and its Member States' other international commitments.

 Point 2. I have expanded the concept of climate justice. In the new version, I begin by distinguishing between isolationist and integrationist methodologies in relation to climate justice. I then argue that a way to make both methodologies less vague and more operational would be to state that the EU's CBAM is inequitable if it poses a significant obstacle to low- and lower-middle-income countries trading with the EU, and if the development of these countries depends on this trade. I emphasised how this reflects the concrete concerns of trading partners in the Global South. I also addressed the issue of the international political acceptability of the CBAM.

 Point 3. I have added and discussed the credit-based approach as a fourth method of navigating the EU's CBAM. This is based on Sandler & Schrag (2025). This makes the literature review proposed by the article more comprehensive and policy-guiding. Thank you so much for this.

 Point 4: I have rewritten the discussion section (Section 4) to incorporate your suggestions. I have clarified which approach dominates over another, depending on the circumstances relating to carbon leakage and fiscal capacity. I have also added some considerations on the credit-based approach (see Point 3). I have added a final paragraph on possible future developments in the CBAM trilemma. These things have also been taken into account in the introduction, where I refer to the proposed amendments to the CBAM regulation put forward by the EU Commission after the first version of the article was published. Finally, I have corrected the citation of Wang (2025). Thank you very much for all this.

# Reviewer report: Reviewer response for version 1

**DOI:** 10.21956/openreseurope.23975.r67577

**Published:** 2026-01-10

**Authors:** Ely Sandler

**Affiliations:** 1 Harvard Kennedy School, Cambridge, Massachusetts, USA

The article frames the implementation of CBAM as a trilemma involving three competing objectives:

1. Level Playing Field
2. Global Climate Justice
3. De Facto Carbon Pricing Club

The article reviews three primary design approaches to navigate this trilemma: a "dual-track" approach (using foreign aid to offset CBAM impacts), exemptions (differentiated pricing for vulnerable countries), and revenue recycling (returning CBAM proceeds to trading partners).

 The article is well-written and researched, as well as scientifically sound and policy-relevant. However, several points should be addressed.

**1. Objectives of the EU vs. Objectives of CBAM**

 The article attributes the "three conflicting policy objectives" to the CBAM itself. Technically, the CBAM itself has a singular objective: addressing the risk of carbon leakage to maintain a level playing field. Objectives such as "climate justice" or "establishing a climate club" are broader EU policy goals, not inherent goals of the CBAM.

 To address this, the title and abstract should be revised to refer to the three conflicting policy goals of the EU in its implementation of CBAM, rather than the goals of the CBAM. 

**2. Credit-Based Approaches to Solving the Trilemma**

 The review of approaches should address a fourth option suggested in the literature whereby exporters to the EU from developing countries can retire carbon credits (e.g., Article 6 credits) up to the value of their CBAM bills. This would involve the EU allowing such retirements to qualify as a "carbon price effectively paid". See Sandler & Schrag (2025) - this approach should be added to the comparative analysis in Section 4 to provide a comprehensive review of the current policy landscape.

**3. Broadening "Climate Justice"**

 While ****the article's addressing of "climate justice" is clear, it may be framed too narrowly, as the EU's broader goal is the political acceptability and trade continuity for developing countries. The primary concern for many global south partners is not just abstract justice, but the practical ability to continue trading with the EU without facing prohibitive barriers that could derail their economic development. To address this, the author could rephrase the "justice" objective as a "trade and political acceptability" objective to make the trilemma more robust for policy analysis.

Are all factual statements correct and adequately supported by citations?

Yes

Is the review written in accessible language?

Yes

Are the conclusions drawn appropriate in the context of the current research literature?

Yes

Is the topic of the review discussed comprehensively in the context of the current literature?

Yes

Reviewer Expertise:

Carbon pricing

I confirm that I have read this submission and believe that I have an appropriate level of expertise to confirm that it is of an acceptable scientific standard, however I have significant reservations, as outlined above.

## References

[1] : Carbon tax assets for carbon tax liabilities: using CBAM to increase climate finance. *Climate Policy* .2025; 10.1080/14693062.2025.2543096 1-11 10.1080/14693062.2025.2543096

# Response

**Published:** 2026-03-09

**Authors:** Fausto Corvino

**Affiliations:** Hoover Chair in Economic and Social Ethics & Insti, Universite catholique de Louvain, Louvain-la-Neuve, Walloon Region, Belgium

Dear Ely Sandler,

 Thank you very much for your report. It was extremely helpful in guiding me through the process of creating a second version of the article. In the new version, I have addressed all of the issues you raised. Specifically, I have made the changes outlined below. Please note that, as some of the issues raised in reports 1 and 2 are similar, some of the responses below are repeated in my responses to report 2 and vice versa. This is unavoidable as I am not allowed to provide a general response to the two reports received so far; I can only respond to them individually.

 Point 1. Throughout the new version, I refer to EU policy goals in the implementation of the CBAM rather than to CBAM objectives. I also clarify that the explicit objective of the CBAM regulation is carbon price equivalence, with the other goals mainly deriving from the EU and its Member States' other international commitments.

 Point 2. I have added and discussed the credit-based approach as a fourth method of navigating the EU's CBAM. This is based on Sandler & Schrag (2025). This makes the literature review proposed by the article more comprehensive and policy-guiding. Thank you so much for this.

 Point 3. I have expanded the concept of climate justice. However, I have not abandoned the more abstract formulation. I have decided to retain both formulations. I started from the distinction between isolationist and integrationist methodologies in relation to climate justice. I then argued that a way to make both methodologies less vague and more operational would be to state that the EU's CBAM is inequitable if it poses a significant obstacle to low- and lower-middle-income countries trading with the EU and if legitimate expectations of development in these countries depend on this trade. I emphasised how this reflects the concrete concerns of trading partners in the Global South. I also addressed the issue of the international political acceptability of the CBAM. Taking all this into account, I have chosen to retain the term 'climate justice goal' to emphasise the connection between the empirical and normative aspects of the article.
